# What of the Potato?

**Published:** 1887-12

**Authors:** 


### WHAT OF THE POTATO?

FROM POPULAR SCIENCE NEWS, BY THE SENIOR EDITOR.

The potato as a natural vegetable product is a monstrosity. It is simply a mass of starch granules grouped together in grotesque and irregular ways, so that protuberances of greater or less size, puffed up into singular forms make up the structure. The potato viewed as an article of food has few nutrient principles to recommend it, but as a table luxury it is highly esteemed in all lands where it has been introduced. What is more appetizing than a healthy potato just from the hot oven, with the pleasant aroma rising with the liberated steam as the outer covering is removed, and with crumbling, snowy-white starch granules falling upon the plate at the breakfast table? But nature never intended that it should subserve a higher purpose in human alimentation than one which is strictly secondary. The high purpose of foods is two-fold: first, to maintain animal warmth; second, to supply the needed amount of energy which every-day labor demands. The waste of the body which is constantly occuring, sleeping or waking, must be met by a supply of food, which contains the chemical principles fitted to replenish and sustain the body. The element nitrogen is the important agent in foods, which supplies force or energy, and this the potato holds only in insignificant quantities. It is capable of supplying warmth to a considerable extent, but it cannot be regarded as a carbonaceous food of high value.

The only people in the world who have fallen into the grievous error of striving to subsist almost entirely upon potatoes, are the Irish. It cannot be doubted that nearly or quite all the ills that trouble unhappy Ireland are due to the humble potato. The Irish are the meanest-fed people living in a civilized land, of which we have any knowledge ; they are in a condition of semi-starvation, even when in their highest prosperity.

Every visitor in Ireland is struck with the "pot-bellied" appearance of the natives, men and women. This abnormal distention of the abdominal walls is due to the enormous amount of potatoes, which they are compelled to consume, in order to maintain a tolerable degree of health. If this people would quit the cultivation of the potato, and supply its place with cereal grains, Ireland would soon become a happy and prosperous nation.

The Irish problem is regarded by English statesmen, as an exceedingly intricate one. It is certainly of a nature which cannot be solved by angry debates in Parliament or by ministerial changes. But if the English landowners will take the trouble to visit Ireland, and remain long enough to change its soil productions from potatoes to cereal grains, Irish riots and discontent will soon cease.

Half-starved men and women can never be happy, and an exclusive diet of potatoes, no matter how large the quantity used, will only serve to maintain people in a feeble, half-starved, revolutionary, quarrelsome condition.

William Cobbet once wrote that the ruin of Ireland would result from the cultivation of that "dirty root," the potato. Adam Smith declared, years ago, that England would some day have to support Ireland, if potato culture was not abandoned for the raising of other soil products. He believed that the same extent of land which would grow food for one person in England, would support four in Ireland. The soil is better, the climate is better ; and the only need is, to throw seed-potatoes into the sea, and plant wheat, oats, barley, and other grains, instead of the innutritious tubers.

Chemical laws, whether those involved in the formation of a world, or those connected with the sustentation and healthy condition of animal life, cannot be violated or ignored, without causing evils of the greatest magnitude. It is well to remember this important suggestion, if we desire to be happy and prosperous.

